# Double Emulsion Microencapsulation System for *Lactobacillus rhamnosus GG* Using Pea Protein and Cellulose Nanocrystals

**DOI:** 10.3390/foods14050831

**Published:** 2025-02-27

**Authors:** Sanket Prakash Vanare, Rakesh K. Singh, Jinru Chen, Fanbin Kong

**Affiliations:** Department of Food Science & Technology, The University of Georgia, 100 Cedar St #211, Athens, GA 30602, USA; sanket.vanare@uga.edu (S.P.V.); rsingh@uga.edu (R.K.S.);

**Keywords:** microencapsulation, probiotics, double emulsion, pea protein, cellulose nanocrystals, in vitro digestion

## Abstract

Microencapsulation using a double emulsion system can improve the viability of probiotic cells during storage and digestion. In this study, a double emulsion system W_C_/O/W_F_ was designed to microencapsulate *Lactobacillus rhamnosus GG* using pea protein (PP) and cellulose nanocrystals (CNCs) at various proportions, and the effect of their proportions on the stability and efficacy of the encapsulation system was studied. The double emulsions were prepared by a two-step emulsification process: the internal aqueous phase containing probiotic strain (W_C_) was homogenized into the oil phase (O), which was then homogenized into the external aqueous phase (W_F_) containing 15% wall materials with varying proportions of PP and CNCs [F1 (100:0), F2 (96:4), F3 (92:8), F4 (88:12), F5 (84:16), F6 (80:20)]. The incorporation of CNCs significantly lowered the average particle size and improved the stability of the emulsions. The encapsulation efficiency did not differ significantly across the tested formulations (63–68%). To check the effectiveness of the designed system, a simulated digestion study was conducted in two phases: gastric phase and intestinal phase. The double emulsion microencapsulation significantly improved the viability of encapsulated cells during digestion compared against free cells. Microscopic analysis along with assessment of protein hydrolysis of the double emulsions during the simulated digestion demonstrated a two-stage protection mechanism. This study presented promising results for employing a double emulsion system for the microencapsulation of probiotics and the potential of PP and CNCs in designing such systems.

## 1. Introduction

Probiotics are live microorganisms that provide numerous health benefits when consumed in sufficient numbers [1]. Probiotics have come under focus in recent years due to the numerous health benefits they offer, not just in terms of digestive health [2,3] but also in the management of infantile colic [4], rheumatoid arthritis [5], obesity [6], diabetes [7], Alzheimer [8], and Parkinson’s [9]. The common probiotic bacteria used in functional foods include species such as *Lactobacillus* spp., *Bifidobacterium* spp., *Streptococcus* spp., *Enterococcus* spp., and *Saccharomyces boulardii* [10]. Probiotics are incorporated into various products, dominantly dairy products, fruit juices, chocolate products, non-dairy drinks, and cereal products [11]. To qualify for health claims, the number of probiotics should be at least 10^6^–10^7^ CFU per gram product [1].

Encapsulation improves the viability of probiotics during storage and digestion processes. Encapsulation may be defined as a process where active ingredients, such as flavors, nutrients, probiotics, or bioactive compounds, are enclosed within a protective coating or matrix. Amongst the different technologies used for encapsulation—such as extrusion, emulsification, spray drying, freeze-drying, complex coacervation, and fluidized bed drying—emulsification technology is employed frequently due to cost, ease of operation, scalability, and the potential to combine it with other technologies such as spray drying [12,13].

A single emulsion system for encapsulation is usually unstable and exhibits poor encapsulation efficiency. In contrast, a double emulsion system (water-in-oil-in-water or oil-in-water-in-oil) has the capability to encapsulate both hydrophilic and hydrophobic compounds and offers better encapsulation efficiency due to its unique protection mechanism of two films and three phases [14]. Furthermore, targeted delayed release of encapsulated cells can be achieved by designing a double emulsion system with appropriate wall materials, such as a protein–polysaccharide system [15].

Plant proteins such as soy protein and pea protein (PP) have gained traction in recent years due to shifting consumer preferences. Legume proteins, viz. PP, are of further interest due to being perceived as ‘hypoallergenic’ compared to dairy and soy proteins [16]. PP demonstrates functional qualities desirable in encapsulating material including good water solubility, stability at high temperatures, and foaming and emulsifying properties [17]. Therefore, it has been studied extensively in recent years for encapsulation of functional compounds such as curcumin, flavonoids, riboflavin, quercetin, essential oils, etc. [18,19,20,21,22,23].

Cellulose nanocrystal (CNC) has recently come into focus for its interesting features, including a large surface area, biocompatibility with a variety of materials, ease of modification, and biodegradability [24]. Due to their surface properties, cellulose nanocrystals act as an excellent dispersant for Pickering emulsions [25,26,27,28]. CNCs have also been found to improve the stability and controlled release of encapsulated probiotics [29,30,31,32,33].

Despite their promising characteristics, pea protein and CNCs have been underutilized in emulsion systems for probiotic encapsulation. There are few studies available employing pea protein in an emulsion encapsulation system for probiotics [34,35,36,37,38,39,40]. Furthermore, there is no literature available on the application of PP and CNCs in a double emulsion microencapsulation system for probiotics, neither individually nor combined.

The aim of this study was to design and characterize a double emulsion system for microencapsulation of *Lactobacillus rhamnosus GG* (LRGG) using PP and CNCs. The physicochemical properties and structural features of the double emulsion systems formed by varying compositions of PP and CNCs were studied. The viability of the LRGG cells during simulated gastrointestinal digestion was measured and compared for all emulsion compositions. In addition, the effect of CNCs on the hydrolysis of protein during digestion stages and hence their effect on the effectiveness of encapsulation system were also studied and discussed.

## 2. Materials

The LRGG cells were purchased as probiotic supplement capsules from Culturelle Probiotics (Shelton, CT, USA). Each capsule contained 10 billion CFUs of LRGG. Difco^TM^ Lactobacilli MRS broth and Bacto^TM^ Peptone were purchased from Becton, Dickinson and Company (Sparks Glencoe, MD, USA). To prepare the oil phase, Great Value vegetable oil was purchased from Walmart (Atlanta, GA, USA). The emulsifier TWEEN^®^80 was purchased from Acros Organics (Milton Park, NJ, USA). The PP used was PURIS^TM^ Pea Protein 870 H (P870H) and was provided by PURIS, LLC (Turtle Lake, WI, USA). The nutrition data and amino acid composition of P870H are provided in Table 1. CNCs (9.5 wt%, 5–20 nm wide, 150–200 nm long) were provided by the Process Development Center at the University of Maine (Maine, USA). For the enumeration of cells, agar plates were prepared using Lactobacilli MRS Agar purchased from Alpha Biosciences (Baltimore, MD, USA). Chemical reagents used for the preparation of simulated gastric fluid (SGF) and simulated intestinal fluid (SIF) were purchased from Sigma-Aldrich Corp. (St. Louis, MO, USA), J. T. Baker Inc. (Phillipsburg, NJ, USA), and Carolina Science (Burlington, NC, USA). The digestive enzymes pepsin from porcine gastric mucosa, pancreatin from porcine pancreas, lipase from porcine pancreas, and bile extract porcine were purchased from Sigma-Aldrich Corp. (St. Louis, MO, USA).

## 3. Methods

### 3.1. Preparation of Double Emulsion W_C_/O/W_F_

To prepare the internal aqueous phase W_C_, the LRGG cells were first incubated in 0.1% peptone water solution (PW) for 24 h at 37.5 °C. Using an inoculation loop, 10 µL of this solution was transferred to 10 mL MRS broth and incubated at 37.5 °C for 24 h. After the incubation, the growth medium containing the cell growth was transferred to a sterile centrifugal tube. The growth medium was centrifuged at 4000 rpm for 10 min. After the centrifugation, the supernatant was discarded. The cells were washed twice with sterile 0.1% PW (10 mL sterile 0.1% PW was mixed with the pellet and then centrifuged). The centrifugation was performed using Hermle Compact Z207A Centrifuge (Hermle Centrifuges USA, Sayreville, NJ, USA). The isolated bacterial cells were then suspended in 100 mL water. The oil phase (O) was prepared by adding TWEEN^®^80 to vegetable oil (2% by volume). The W_C_ suspension was slowly added to the oil phase with constant stirring using a magnetic stirrer for 10 min (40:60 by volume). The primary emulsion W_C_/O was prepared by homogenizing the mixture using a rotor-stator homogenizer (Bamix, Biospec Products, Bartlesville, OK, USA) at 10,000 rpm for 5 min.

**Figure 1:**
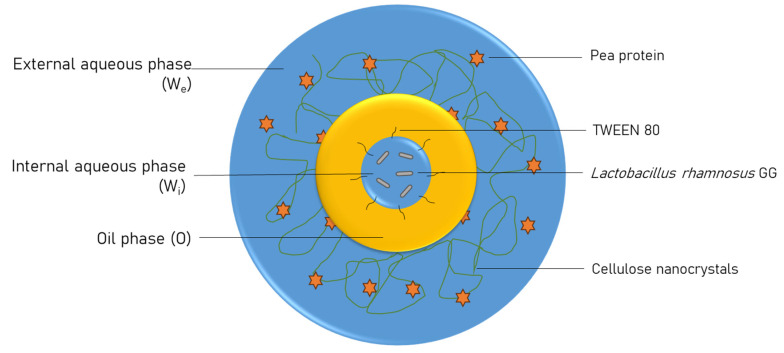
Graphical representation of double emulsion system microencapsulating *Lactobacillus rhamnosus* GG cells using pea protein and cellulose nanocrystals.

The outer aqueous phase consisting of wall materials W_F_ was prepared using PP and CNCs. The wall materials were added into the water at a concentration of 15% *w*/*v* and stirred for 15 min using a magnetic stirrer. Six formulations of wall material were studied with varying proportions of PP and CNCs (Table 2). The highest level of CNC incorporation was decided from preliminary work. At concentrations of CNCs higher than 20% in the wall material, the external aqueous phase W_F_ demonstrates gelation, which is undesirable in the formation of a homogenous double emulsion. The primary emulsion W_C_/O was homogenized into W_F_ (40:60 by volume) at 5000 rpm for 2 min to prepare the double emulsion W_C_/O/W_F_. Figure 1 demonstrates a pictorial representation of the double emulsion W_C_/O/W_F_.

### 3.2. Particle Size Analysis

The De Brouckere mean diameter D[4,3] of the W_C_/O/W_F_ double emulsions was measured using a laser diffraction particle size analyzer (Mastersizer MSS 5004, Malvern Panalytical, Westborough, MA, USA). The samples were dispersed in a measurement chamber with deionized water to achieve an obscuration of 12–15%. The analyzer was equipped with a He/Ne laser (5 mW, 300 RF). All measurements were repeated at least three times at ambient temperature.

### 3.3. ζ-Potential

To study the stability of the double emulsions, the ζ-potential of the W_C_/O/W_F_ double emulsions was measured using a particle size analyzer upgraded for ζ-potential measurements (Brookhaven 90Plus particle size analyzer, Brookhaven Instruments, Nashua, NH, USA). The analysis was performed with a solid-state laser (35 mW, standard) at scattering angles of 15° and 90°. The samples were diluted with DI water at 1% concentration to achieve optimal signal. All measurements were repeated at least three times.

### 3.4. Optical Microscopy

The morphology of the W_C_/O/W_F_ double emulsions was studied using a microscope (Nikon Eclipse E600 Microscope, Nikon Metrology Inc., Brighton, MI, USA). The light source used was 12 V 100 W Halogen, with a 100 W Mercury Lamphouse fluorescence attachment. The samples were diluted at 25% *v*/*v* with DI water to achieve optimum resolution. The diluted samples were observed using a 10X eyepiece and 40X objective lens.

### 3.5. FT-IR

The structural analysis of the W_C_/O/W_F_ double emulsions was assessed using FT-IR spectroscopy. The FT-IR spectra of the PP, CNC, and W_C_/O/W_F_ double emulsions were obtained using an FT-IR spectrometer (Nicolet 6700 FT-IR, Thermo Electron Corp., Waltham, MA, USA). The FT-IR spectrum was collected in the wavenumber range of 4000 to 400 cm^−1^ and compared for the wall materials and the double emulsion.

### 3.6. Encapsulation Efficiency

The encapsulation efficiency (EE) of LRGG in W_C_/O/W_F_ double emulsion was calculated using the methods described in the literature [15,41,42] with some modifications. The W_C_/O/W_F_ double emulsions with encapsulated LRGG cells were centrifuged at 13,500× *g* for 10 min. The encapsulated cells were liberated due to the centrifugation. The total number of cells was then calculated using the standard plate method. The broken emulsion samples were serially diluted using 0.1% PW and plated on Lactobacilli MRS agar plates. The colonies were counted after incubation at 37 °C for 48 h. The viable cell count in the whole double emulsion was calculated by this method, denoted by N_T_. The unencapsulated cells in the emulsion were counted by directly diluting the double emulsions, denoted by N_F_. The EE was calculated using the following formula:

$$\mathrm{EE}\;\%=\frac{N_{T}\;\left(\mathrm{CFU}/\mathrm{mL}\right)-N_{F}\;\left(\mathrm{CFU}/\mathrm{mL}\right)}{N_{T}\;\left(\mathrm{CFU}/\mathrm{mL}\right)}\times 100$$

### 3.7. In Vitro Digestion

The static in vitro digestion study of the W_C_/O/W_F_ double emulsions was performed to study the viability of encapsulated LRGG cells during a simulated digestion process. The digestion processes were designed and conducted as per standard methods [43].

The digestion was carried out in two phases: gastric digestion and intestinal digestion. The emulsion samples and SGF were mixed in a ratio of 5:4 by volume. Porcine pepsin was added to the mix to achieve an activity of 2000 U/mL in the digesta, followed by CaCl_2_ for a desired concentration of 0.075 mM in the digesta. The pH was adjusted at pH 2 using 1 M HCl. The digestion was carried out in a shaking hot tub at 37 °C (Boekel Scientific, Feasterville-Trevose, PA, USA). The gastric digestion was carried out for two hours. An amount of 1 ml digesta sample was drawn after each 30 min for further analysis, and the digesta was replenished with 1 mL SGF. The pH of the digesta was adjusted to pH 2 every 30 min, and the amount of HCl required was used to measure the extent of protein hydrolysis.

After 2 h of gastric digestion, the gastric chyme was mixed with SIF in the ratio of 5:4 by volume. Pancreatin was added to the digesta to achieve a trypsin activity of 100 U/mL. Since the samples contained high amounts of fats, lipase was added to the digesta to achieve a final activity of 2000 U/mL. Additionally, CaCl_2_ was added at 0.3 mM concentration in the digesta. The pH was adjusted to pH 7 using 1 M NaOH. The intestinal phase was carried out for 1 h; 1 mL digesta sample was drawn every 30 min and replenished with 1 mL SIF. The pH was adjusted every 30 min, and the amount of HCl required was used to measure the extent of protein hydrolysis.

The 1 mL digesta samples drawn every 30 min were serially diluted with 0.1% PW and plated on Lactobacilli MRS agar plates to enumerate the number of viable cells throughout the simulated digestion process.

Samples were also drawn at each stage of the in vitro digestion study and observed using microscopy to study the morphological changes in the emulsion structure.

### 3.8. Protein Hydrolysis

The external aqueous phase W_F_ is the first phase of the double emulsion system to undergo digestion. The digestion of PP, which is the major component of the W_F_, will determine the length of the exposure of the primary emulsion to the gastric and intestinal digestion, and hence the effectiveness of the delivery system. Hence, to estimate the potential gastric fate of the W_C_/O/W_F_ double emulsions, the digestion of PP in the form of degree of protein hydrolysis during the digestion stages was studied. The degree of protein hydrolysis (DH) was analyzed using the pH-stat method [44,45,46,47]. Specifically, the amount of HCl solution required to maintain the pH of the digesta in respective phases was recorded over the digestion time. The DH was then calculated using the following equation:

$$\mathrm{DH}=\left[\frac{\left(V_{A}\times {\;N}_{A}\right)}{M_{P}}\right]\times \left(1/P\right)\times F_{\mathrm{PH}}\times 100$$

Here, V_A_ is the volume of HCl required (ml), N_A_ is the normality of HCl (1 N), M_P_ is the mass of the protein in the digesta (varied according to the formulation), P is the total number of peptide bonds (7.55 mol/g for PP) [47], and F_PH_ is the correction factor that accounts for the partial ionization of peptides [44,45,46].

### 3.9. Statistical Analysis

Experimental data were analyzed using the ANOVA (analysis of variance) approach with level of significance at *α* = 0.05. R Studio (version 2024.09.1) was used as the software for statistical analysis.

## 4. Results and Discussion

### 4.1. Droplet Size

Many physiochemical properties of emulsions, including stability, depend on the droplet size of emulsion droplets. Hence, it is important to assess the droplet sizes of the double emulsion microencapsulation systems. The average De Brouckere mean diameter for each formulation of the W_C_/O/W_F_ double emulsion is listed in Table 3. The range of the emulsion droplet sizes confirmed that the type of encapsulation achieved for the LRGG cells was microencapsulation. There was a significant difference (*p* < 0.05) between the droplet sizes of the six double emulsions, negatively correlated with the proportion of CNCs in the matrix. The average droplet size of the emulsions without CNCs was significantly larger than the emulsions with a higher proportion of CNCs in the encapsulation matrix (12%, 16%, and 20%) (see Supplementary Materials). None of the emulsions with CNCs differed significantly in their average droplet size.

A similar trend was observed in a previous study focusing on the effect of CNCs on pea protein morphology and gel properties [48]. A possible explanation is that CNCs stabilize the dispersion of PP molecules in the emulsion system which inversely affects the aggregation of large PP molecules. Furthermore, the addition of CNCs to the external aqueous phase W_F_ leads to a more compact and stable structure of CNCs and PP, thus reducing the extent of protein aggregation and lowering the average droplet size [49]. In addition, the high viscosity of the emulsion system due to addition of CNCs also hinders the aggregation of PP molecules [49]. The stabilizing effect of CNCs on the double emulsion system is further discussed in Section 4.2.

### 4.2. Emulsion Stability

ζ-potential is an indicator of the charge distribution of the particles in a dispersion system. If the absolute value of ζ-potential is low, that means there is not enough repulsive charge between the microparticles and the system is not stable, i.e., the attractive forces overcome the repulsive forces between the suspension particles. A ζ-potential value greater than +30 mV or lower than −30 mV is considered to imply a stable emulsion system [50].

The ζ-potential values for the W_C_/O/W_F_ double emulsion systems are enlisted in Table 3. The inclusion of CNCs in the encapsulation matrix had a significant effect on the stability of the double emulsions. The emulsion formulated without CNCs had a significantly lower ζ-potential value than the emulsions with CNCs (*p* < 0.05). The emulsions with CNCs had a ζ-potential value of lower than −30 mV and yielded a stable double emulsion system (see Supplementary Materials).

The results agree with previous findings, wherein CNCs have been shown to improve the stability of CNC–protein systems significantly [51]. There are various mechanisms proposed in the literature for the stabilization effect of CNCs in an emulsion system containing proteins. In a previous study concerning the effect of CNCs on the gelling properties of pea protein isolates (PPIs), it was observed that the PPI molecules formed an orderly network in the presence of CNCs and decreased the irregularity of protein aggregation [48]. CNCs were found to bind to the amino acids legumin and vicilin present in the PP via hydrogen bonding and van der Waals bonding. Another possible mechanism for the stabilization effect of CNCs might be due to the adsorption of CNCs on the interface of the emulsion droplets, hence reinforcing the proteins on the interface. The non-adsorbed CNC molecules then interact in the continuous phase, increasing the system viscosity and contributing to the stability [52].

### 4.3. Optical Microscopy

**Figure 2:**
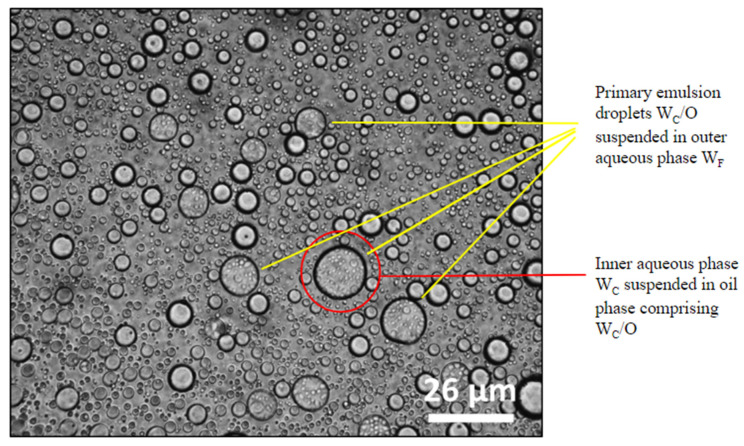
Optical microscopy pictures of double emulsion encapsulation system (formulation F2) of *Lactobacillus rhamnosus GG* using pea protein and cellulose nanocrystals.

The microstructures of the W_C_/O/W_F_ double emulsions can be observed in the images taken using optical microscopy (Figure 2). The double emulsion droplets can be observed to be spherical and intact, similar to other studies involving double emulsions observed using optical microscopy [14,41]. The encapsulation can be inferred to be of polynuclear type since multiple primary droplets are observed within the secondary droplets [53]. The double emulsions were also observed using optical microscopy during different stages of simulated digestion, the results of which are discussed in Section 4.7.

### 4.4. FT-IR Analysis

**Figure 3:**
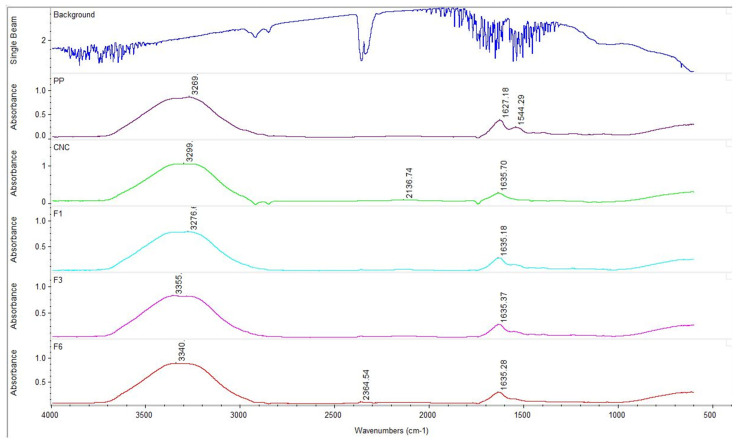
Fourier-transform infrared spectroscopy of pea protein, cellulose nanocrystals, and double emulsions microencapsulating *Lactobacillus rhamnosus GG* using pea protein and cellulose nanocrystals (formulations F1, F3, and F5).

FT-IR spectroscopy was used to study the interactions between PP and CNCs in the W_C_/O/W_F_ double emulsions. Figure 3 demonstrates the FT-IR spectrum for PP, CNCs, and double emulsions with formulations F1, F3, and F6. The broad peak in the range of 3500–3000 cm^−1^ is associated with stretching vibrations of O-H and CH_2_-OH groups overlapping with the primary and secondary amine groups of PP. The peaks at 1627 cm^−1^ in PP and 1635 cm^−1^ in CNCs and double emulsions correspond to stretching vibrations of C=C groups. The peaks observed at 1635 cm^−1^ in all spectra correspond to C=C stretching bands (alkenes). Pure PP shows a distinct peak at 1544 cm^−1^ not observed in CNCs or emulsions. This peak corresponds to N-H stretching bands (amide III). It can be inferred that some of the protein–protein interactions present in the PP are replaced with interactions between protein and CNCs in the double emulsions.

### 4.5. Encapsulation Efficiency

The encapsulation efficiencies (EEs) of all the W_C_/O/W_F_ double emulsions are listed in Table 3 and in Supplementary Materials. The EE of the double emulsions did not differ significantly (*p* = 0.81), meaning the EE of the microencapsulation system was not affected by the proportion of CNCs in the encapsulation matrix. This might be due to several reasons. The EE of a double emulsion encapsulation system depends mainly on the stability of the primary emulsion since the probiotic cells are enclosed within the primary emulsion. The surfactant used in the primary emulsion W_C_/O was TWEEN^®^80, a hydrophilic nonionic surfactant with a high HLB value. TWEEN^®^80 has been observed as an effective stabilizer for a W/O/W emulsion system when combined with protein [54]. In addition to TWEEN^®^80, both PP and CNCs have emulsifying abilities that contribute to the stability of the encapsulation system [28,55].

### 4.6. Protein Hydrolysis

The digestion of the protein throughout the gastric and intestinal digestion of the W_C_/O/W_F_ double emulsions was assessed using the pH-stat method. This method is based on the principle that the pH change occurring in the digestive media during digestion is due to the cleavage of peptide bonds, i.e., protein hydrolysis. This pH change is reflected in the amount of acid or base required to keep the pH constant during digestion.

**Figure 4:**
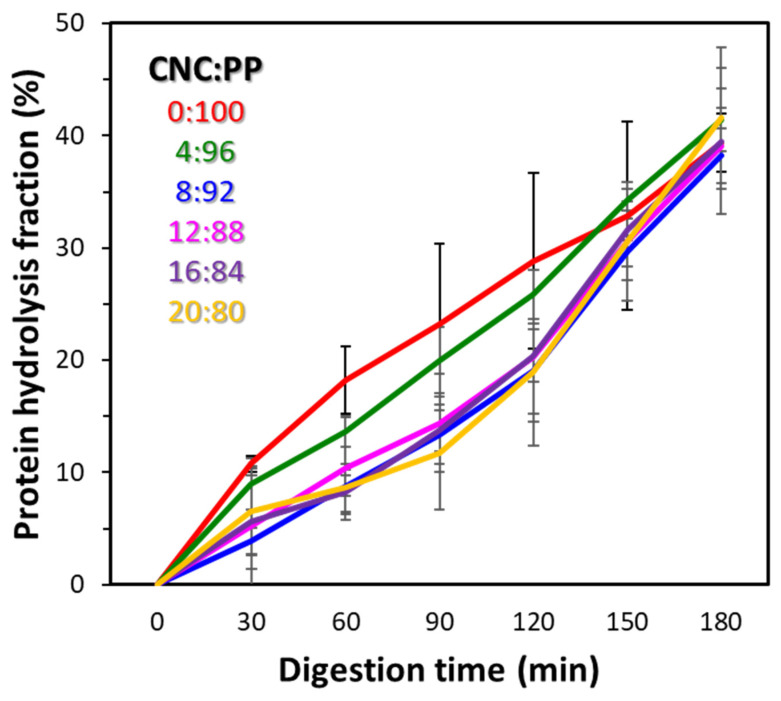
The protein hydrolysis (%) of PP in different formulations of double emulsions microencapsulating *Lactobacillus rhamnosus GG* in a simulated gastrointestinal digestion model. (0–120 min: gastric digestion; 120–180 min: intestinal digestion).

The protein hydrolysis fraction of all the emulsions is demonstrated in Figure 4. The double emulsion without CNCs showed a fast rate of hydrolysis in the initial stages of the digestion and then slowed down in the later stages. The double emulsion with 4% CNCs in the encapsulation matrix showed a similar trend. In contrast, emulsions with a higher proportion of CNCs in their wall matrix showed a slow hydrolysis rate initially but reached a similar extent of hydrolysis by the end of the intestinal stage. These observations might be due to the effect of CNCs on the digestion of proteins in CNC–protein composites [56]. At higher incorporation levels, CNCs can restrict the ability of pectic enzymes to come into contact with PP during the initial stage of gastric digestion. With time, the physical force due to the constant mixing as well as a high acid environment weakens the emulsion stability allowing the digestive enzymes to access the protein molecules. Furthermore, denatured proteins are susceptible to a faster rate of hydrolysis compared to native proteins [46]. Hence, in the later stages of digestion, once the proteins are exposed to the gastric conditions and their denaturation is initiated, the hydrolysis rate accelerates.

The relatively low extent of protein hydrolysis observed for all double emulsions is similar to other in vitro studies in the literature [45,46]. This might be due to the lack of complexities of in vivo digestion processes, such as peristaltic moments, in a static digestion model. Hence, digestion studies of microencapsulation systems in a dynamic digestion model can report more accurate information compared to static models [57,58].

### 4.7. In Vitro Simulated Digestion

The total number of viable cells of LRGG during the simulated gastrointestinal digestion study is tabulated in Table 4.

During the digestion of the control, i.e., free cells, no viable cells were detected after 60 min at pH 2.0, similar to other studies in the literature [30]. This further emphasizes the importance of microencapsulation of probiotics to achieve targeted delivery in the intestinal tract. In contrast to the control, the microencapsulated cells showed excellent viability, with the W_C_/O/W_F_ double emulsions achieving a log reduction of about 3 log CFU on average over the complete digestion process. The log reduction values in CFUs after digestion were not significantly different for the double emulsion systems; however, the incorporation of CNCs was observed to improve the cell viability with increasing levels. This observation aligns with prior studies incorporating CNCs in the encapsulation system for probiotics. CNCs were reported to decrease gastric fluid absorption in a digestive study of alginate–CNC–lecithin-based microencapsulation system for probiotics, hence better survivability [59]. The statistical insignificance might be due to the low number of initial CFUs in the samples, and at a higher number of probiotic encapsulation such as in industrial applications, a significant difference may be observed.

**Figure 5:**
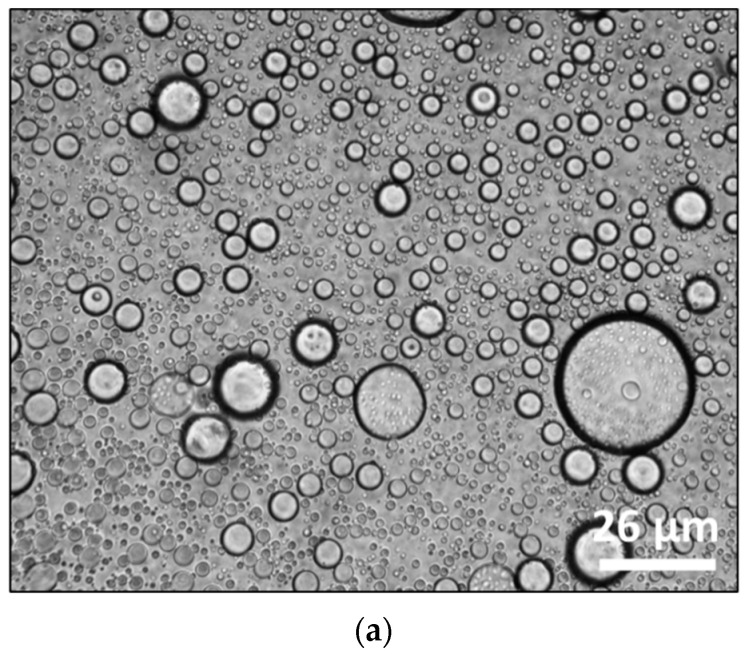

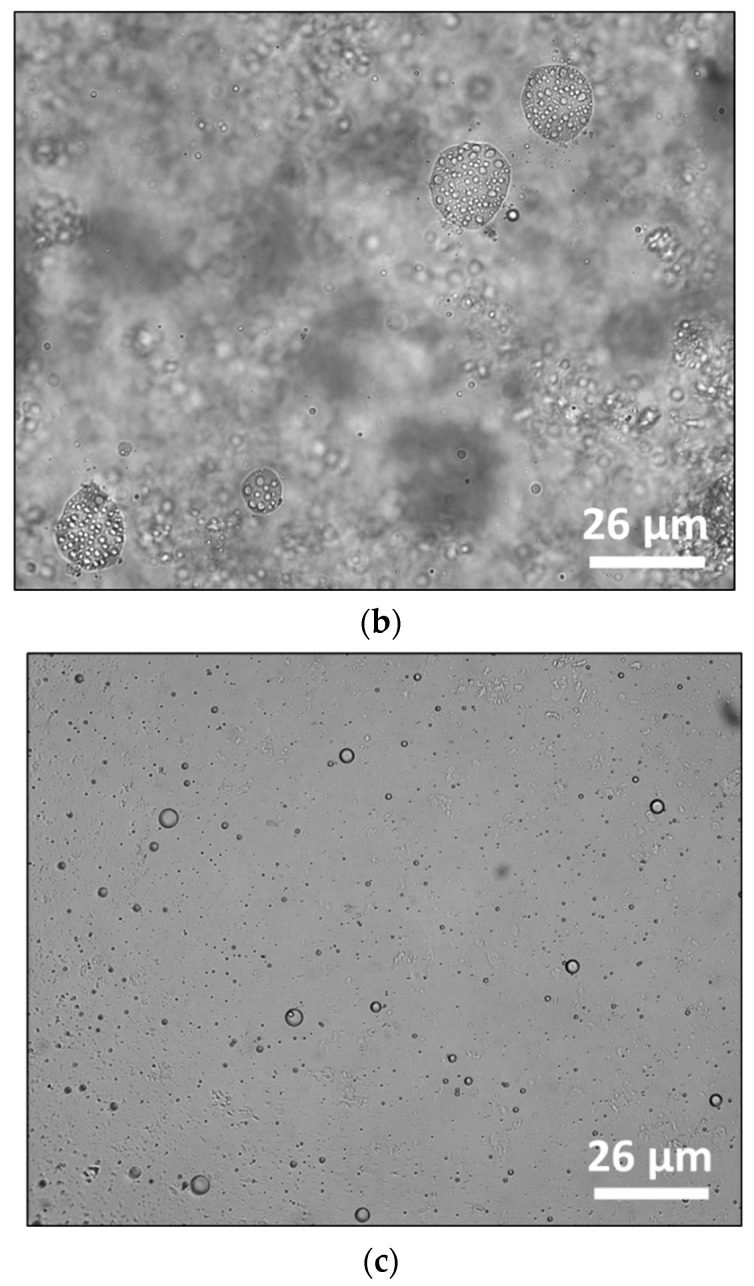
Optical microscopy images of double emulsion system (formulation F2) prepared using pea protein and cellulose nanocrystals microencapsulating *Lactobacillus rhamnosus GG* during simulated digestion study (**a**) before digestion, (**b**) after gastric digestion, and (**c**) after intestinal digestion.

Overall, a sharp reduction in the viable cells was observed after the first 30 min. This loss most probably corresponds with the free cells in the double emulsions. After this stage, the cells maintain a slow steady decrease in viability during the gastric stage. This period might correspond to a gradual weakening of the emulsion system, as observed in the protein hydrolysis assessment, and the subsequent exposure of primary emulsion to the gastric chyme. These observations are supported by gastric digestion studies of double emulsion systems found in the literature [15]. However, the lipolytic enzymes are not added until the intestinal stage; hence, the primary emulsions might still retain some integrity during the gastric phase [43]. This possibility is further corroborated by the microscopic observations of the gastric chyme. At the start of the digestion, intact and uniformly distributed double emulsion droplets can be observed in the system (Figure 5a). After gastric digestion, the emulsion system is not intact; however, some aggregations of primary emulsion droplets can still be observed (Figure 5b). After the intestinal stage, when the lipolytic enzymes are introduced in the gastric chyme, no emulsion droplets can be observed (Figure 5c). At this stage, the lipases can be assumed to access the oil phase of the primary emulsion, breaking it down and releasing the internal phase with probiotic cells [43]. During the intestinal stage, no loss in viability is observed and the number of viable cells remains almost constant. This can be attributed partly to the incorporation of CNCs in the encapsulation system. Polysaccharides have been reported to improve the survival of probiotic cells in the gastrointestinal conditions [12,34,36]. Furthermore, CNCs have been observed to improve the transition of viable cells in simulated gastrointestinal conditions. CNCs improve the hydration of biopolymers during the gastrointestinal processes and help the release of cells in the intestinal phase [59].

### 4.8. Proposed Mechanism for Digestion of PP-CNC Double Emulsions Microencapsulating LRGG

**Figure 6:**
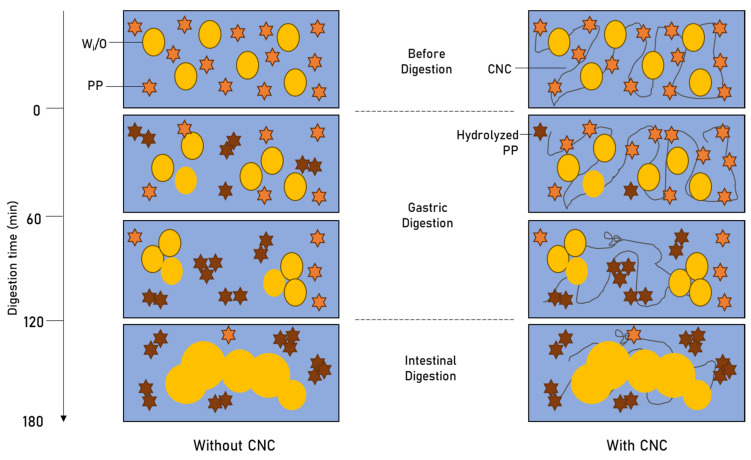
Proposed mechanism for the digestion of double emulsion microencapsulation systems of *Lactobacillus rhamnosus GG* using pea protein and cellulose nanocrystals. The yellow stars represent pea protein, whereas the brown stars represent hydrolyzed pea protein. The yellow circles represent primary emulsion droplets. The swirly lines represent the CNC network.

Taking into account the inferences from all the analyses, a possible mechanism for the double emulsion systems microencapsulating LRGG cells can be put together (Figure 6).

In the absence of CNCs, the secondary emulsion system is unstable and the PP starts to aggregate before the digestion itself, as inferred from the ζ-potential analysis. Once the gastric digestion is initiated, the proteolytic enzymes can access the PP molecules easily, and the protein hydrolysis starts immediately, as seen in the protein hydrolysis study (Figure 4). The gastric pH of 2 contributes to the denaturation and hence accelerated hydration of PP [46]. However, at the end of the gastric digestion, the primary emulsion still retains some integrity, as observed using optical microscopy (Figure 5b). In the intestinal phase, lipase is introduced, which then initiates the digestion of the oil phase in the primary emulsion droplets, releasing the inner aqueous phase containing the LRGG cells.

In the presence of CNCs, the double emulsions are stable with a uniform PP-CNC network [48,51,52]. As the digestion starts, the PP molecules are initially shielded from the gastric enzymes due to the stabilizing effect of CNC molecules [56]. With time, due to the physical mixing and the gastric pH of 2, the network weakens, and the hydrolysis of PP accelerates during the second hour of gastric digestion (Figure 4). In the intestinal phase, the oil phase of the primary emulsion starts to break down and the encapsulated probiotic cells are released (Figure 5c).

## 5. Conclusions

The encapsulation of probiotic cells significantly improves their stability during storage and digestion processes. In this study, *Lactobacillus rhamnosus GG* cells were microencapsulated in a double emulsion system W_C_/O/W_F_ containing PP and CNCs in the outer encapsulation phase W_F_ in varying proportions. CNCs significantly improved the stability of the double emulsions. All formulations had a high encapsulation efficiency and significantly improved the viability of the cells during the simulated gastrointestinal digestion study. This study showed the effectiveness of a double emulsion system in improving the survivability of probiotics during digestive processes by offering multiple-layer protection. Double emulsion systems can be efficiently designed for targeted delivery of probiotics to the intestinal stages with optimization of the wall materials and the phases. The results also showed the potential of PP and CNCs in designing and stabilizing such encapsulation systems for practical applications. These results are promising for further applications of microencapsulated probiotics in a variety of food and pharmaceutical products.

## Figures and Tables

**Table 1:** (**a**) Nutrition data and (**b**) amino acid composition of PURIS^TM^ Pea Protein 870 H.

**a**			
**Nutrient**	**Values (Per 100 g)**	**Nutrient**	**Values (Per 100 g)**
Moisture (g)	6.0	Carbohydrates (g)	4.0
Protein (dry matter basis) (g)	80.0	Sugars (g)	0.0
Total fat (g)	6.0	Dietary fiber (g)	4.0
Ash (g)	5.0	Calories (kcal)	390.0
Sodium (mg)	750.0	Phosphorous (mg)	1100.0
Potassium (mg)	200.0	Calcium (mg)	400.0
Iron (mg)	10.0	Vitamin D (mg)	0.0
**b**			
**Essential Amino Acids**	**Values (g/100 g)**	**Non-Essential Amino Acids**	**Values (g/100 g)**
Arginine	6.84	Alanine	3.44
Histidine	1.99	Aspartic Acid	9.42
Isoleucine	4.05	Cysteine	0.85
Leucine	6.80	Glutamic Acid	13.32
Lysine	6.11	Glycine	3.28
Methionine	0.85	Serine	3.88
Phenylalanine	3.86	Tyrosine	3.86
Threonine	3.05	Proline	3.55
Valine	4.28		
Tryptophan	0.79		

**Table 2:** Composition of the external aqueous phase (W_F_) of double emulsion encapsulation systems with varying proportions of pea protein (PP) and cellulose nanocrystals (CNCs) for encapsulation of *Lactobacillus rhamnosus GG*.

	Wall Material Composition		Concentration in W_F_		Concentration in W_C_/O/W_F_	
Formulation	CNC %	PP %	CNC %	PP %	CNC %	PP %
F1	0	100	0	15	0	9
F2	4	96	0.6	14.4	0.36	8.64
F3	8	92	1.2	13.8	0.72	8.28
F4	12	88	1.8	13.2	1.08	7.92
F5	16	84	2.4	12.6	1.44	7.56
F6	20	80	3	12	1.8	7.2

**Table 3:** Characteristics of double emulsion encapsulation systems for *Lactobacillus rhamnosus GG* with pea protein (PP) and cellulose nanocrystals (CNCs) as the wall materials. Same superscripts indicate that the values are not significantly different at *α* = 0.05.

Sample	Wall Material Composition (%)		Particle Size D [4,3] (µm)	Encapsulation Efficiency (%)	ζ-Potential (mV)
	PP	CNC			
F1	100	0	10.49 ± 1.76 ^a^	63.0 ± 0.07	−19.23 ± 4.19 ^a^
F2	96	4	7.79 ± 1.33 ^a,b^	62.6 ± 0.06	−30.63 ± 4.95 ^b^
F3	92	8	7.33 ± 1.07 ^a,b^	66.9 ± 0.07	−33.38 ± 5.22 ^b,c^
F4	88	12	7.22 ± 0.89 ^b^	67.6 ± 0.10	−37.25 ± 1.54 ^b,c,d^
F5	84	16	7.06 ± 1.26 ^b^	67.7 ± 0.04	−39.97 ± 3.52 ^b,c,d^
F6	80	20	6.10 ± 0.36 ^b^	68.3 ± 0.02	−46.38 ± 1.38 ^d^

**Table 4:** Total number of viable *Lactobacillus rhamnosus GG* cells (log CFU) during simulated gastrointestinal digestion study of W_C_/O/W_F_ double emulsion microencapsulation system prepared using pea protein and cellulose nanocrystals.

PP:CNC Proportion in Wall Matrix	Time (min)							Total Log Reduction
	Gastric Stage					Intestinal Stage		
	0	30	60	90	120	150	180	
0:100	8.12 ± 0.19	5.69 ± 1.09	5.19 ± 0.36	4.76 ± 0.48	4.67 ± 0.61	4.86 ± 0.04	4.86 ± 0.42	3.26 ± 0.28
4:96	8.23 ± 0.09	6.46 ± 1.90	5.81 ± 1.76	4.84 ± 1.00	3.89 ± 0.54	4.98 ± 0.34	5.20 ± 0.33	3.03 ± 0.27
8:92	8.26 ± 0.29	6.65 ± 2.00	5.89 ± 2.38	5.60 ± 2.15	5.44 ± 1.53	5.94 ± 1.31	5.95 ± 1.24	2.31 ± 0.95
12:88	8.23 ± 0.18	6.98 ± 1.39	6.10 ± 1.98	5.83 ± 1.84	5.06 ± 1.46	5.44 ± 0.40	5.51 ± 0.60	2.71 ± 0.43
16:84	8.21 ± 0.26	7.09 ± 0.86	6.01 ± 1.04	5.17 ± 0.32	4.97 ± 0.40	5.62 ± 0.38	5.71 ± 0.50	2.49 ± 0.25
20:80	8.20 ± 0.23	7.16 ± 0.91	5.75 ± 0.57	5.05 ± 0.37	4.62 ± 0.55	5.91 ± 0.36	6.02 ± 0.55	2.18 ± 0.31

*p* = 0.112.

## Data Availability

The original contributions presented in this study are included in the article/Supplementary Materials. Further inquiries can be directed to the corresponding author.

## Supplementary Materials

The following supporting information can be downloaded at: https://www.mdpi.com/article/10.3390/foods14050831/s1.
